# Is There an Ideal Concentration of Ozonized Oil for the Prevention and Modulation of Zoledronate-Induced Mandibular Osteonecrosis? A Study on Senescent Rats

**DOI:** 10.3390/jfb15120353

**Published:** 2024-11-21

**Authors:** Mirela Caroline Silva, Izabela Fornazari Delamura, Maria Eloise de Sá Simon, Stefany Barbosa, David Tawei Ting, Karen Bechara, Jamil Awad Shibli, Carlos Fernando Mourão, Ana Paula Farnezi Bassi, Edilson Ervolino, Leonardo Perez Faverani

**Affiliations:** 1 Department of Diagnosis and Surgery, Araçatuba School of Dentistry, Sao Paulo State University (UNESP), Araçatuba 16015-050, Sao Paulo, Brazil; mirela.c.silva@unesp.br (M.C.S.); izabela.delamura@unesp.br (I.F.D.); eloise.simon@unesp.br (M.E.d.S.S.); stefany.barbosa@unesp.br (S.B.); ana.bassi@unesp.br (A.P.F.B.); 2 Dental Research Division, Department of Periodontology and Oral Implantology, Federal Fluminense Univesity, Niteroi 21941-617, Rio de Janeiro, Brazil; tingtawei@gmail.com; 3 Dental Research Division, Department of Periodontology and Oral Implantology, University of Guarulhos (UnG), Guarulhos 07115-230, Sao Paulo, Brazil; kbechara@gmail.com (K.B.); jshibli@ung.br (J.A.S.); 4 Department of Basic and Clinical Translational Sciences, Tufts University School of Dental Medicine, Boston, MA 02111, USA; 5 Department of Basic Sciences, Araçatuba School of Dentistry, Sao Paulo State University (UNESP), Araçatuba 16015-050, Sao Paulo, Brazil; e.ervolino@unesp.br; 6 Department of Oral Diagnosis, Division of Oral and Maxillofacial Surgery, Piracicaba Dental School, University of Campinas (UNICAMP), Piracicaba 13414-903, Sao Paulo, Brazil; 7 OMFS, School of Dentistry, São Paulo State University, Araçatuba 16015-050, Sao Paulo, Brazil

**Keywords:** ozone, osteonecrosis, osteonecrosis associated with bisphosphonates, healing

## Abstract

This study aimed to identify whether there is an ideal concentration for applying ozonized oil (OZ) in the post-exodontic alveoli of senescent rats treated with zoledronate (ZOL). Thirty-five female rats, aged 18 months, were divided into five groups: ZOL; ZOL+OZ500; ZOL+OZ600; ZOL+OZ700; and SAL. The groups treated with ZOL, and other concentrations of OZ received applications at a dose of 100 μg/kg, while the SAL group received saline. After three weeks of ZOL application, the animals underwent extraction of the lower first molar. Subsequently, local therapies were initiated: group ZOL+OZ500 at 500 mEq/kg; ZOL+Z600 at 600 mEq/kg; and ZOL+OZ700 at 700 mEq/kg at baseline, and on days 2 and 4 post-operation. Euthanasia was performed on day 28. The microtomographic parameter of bone volume and histometric data on the area of neoformed bone (NFBT) showed the highest values for the ZOL+OZ600 group (*p* < 0.05). All OZ groups had smaller areas of non-vital bone than the ZOL group (*p* < 0.05). The clinical appearance of the operated region showed the alveoli covered with soft tissue, particularly in the OZ groups. All the tested concentrations of OZ were able to prevent and modulate MRONJ. As it presents a greater amount of NFBT, the concentration of 600 mEq/kg seems to be ideal.

## 1. Introduction

With the aging of the population and the increase in metabolic changes in bone tissue, the imbalance in bone turnover is evident. This causes an increase in osteoclastic activity and a decrease in osteoblastic activity [1,2,3]. In relation to the main changes that affect bone tissue, such as osteopenia, osteoporosis, and bone metastases, the most used medications are anti-resorptive drugs like zoledronate, which acts by inhibiting osteoclastic activity and limiting the response of bone tissue to aggression [1,2,3,4,5,6,7,8].

Patients with a history of taking antiresorptive drugs or a history of irradiation in the head and neck region have either exposed bone or bone that can be probed through an intraoral fistula. They are diagnosed with drug-induced osteonecrosis of the jaw (MRONJ) [9], a debilitating condition reported by patients undergoing treatment with these medications. It is associated with delayed bone repair, infection and bone necrosis, thus directly affecting the patient’s quality of life [1,2,3,4,5,7,10,11,12,13].

Numerous treatments have been employed to optimize the bone metabolism, including medicinal ozone [2,9,14,15,16,17,18,19,20,21,22,23,24]. Its oily form has been the subject of studies on wound healing, due to the substance’s role in generating growth factors and local antioxidant action, promoting tissue repair [15,20,21,22].

Studies carried out by Xiao et al. (2017) [22] and Monteiro et al. (2021) [20] on the use of ozonized oil to prevent injuries in cases of MRONJ has shown that this therapy accelerates the alveolar repair process through the regulation of fibroblast function by increasing critical genes, including type I collagen. This has led to positive results in the histological analysis of bone tissue.

Moreover, ozone diluted in oil acts on the tissue through the formation of free oxygen radicals and bioactive products. The latter, in turn, increase the synthesis of growth factors and cell proliferation resulting from the activation of inflammatory responses [18,25].

Although the medicinal use of ozone has been the subject of few studies to date, it has shown promising results in improving bone metabolism and antioxidant activity, modulating immunological responses, and increasing the release of growth factors [15,18,20,21,22,23,24]. These results corroborate prior results from the group itself [11], notwithstanding the fact that these were administered intraperitoneally, a methodology that makes clinical application difficult.

In the absence of well-established protocols for the prevention and treatment of MRONJ and a lack of clinical consensus on the application of ozonized oil in these conditions, the objective of this study aimed to determine the ideal concentration of ozonized sunflower oil (OZ) for use in the post-extraction alveoli of senescent rats treated with zoledronate (ZOL).

The extraction of lower molars in senescent rats was proposed in order to create a critical situation in the post-exodontic socket to reproduce MRONJ. Next, the effect of the aforementioned local therapies was analyzed over a period of 28 days postoperatively.

## 2. Materials and Methods

### 2.1. Animals

A total of 35 female Wistar rats, aged 18 months and weighing 300–350 g, were used in this study. All experimental protocols received approval from the Animal Use Ethics Committee (CEUA) of the Dentistry School of Araçatuba, case No. 0469-2023.

The animals were randomly distributed into 5 groups of 7 rats each. Group 1, the SAL group, served as a positive control and received an intraperitoneal injection of saline solution (0.9% NaCl) every 3 days for 7 weeks. This intervention aimed to produce a stress level comparable to that of the other experimental groups, allowing for a fair comparison across conditions. This group demonstrates the physiological response of animals not treated with zoledronate, providing a baseline reference for assessing the effectiveness of the therapies tested in this research. Group 2 (ZOL), with osteonecrosis induced by the application of ZOL 100 μg/kg, every 3 days for 7 weeks; group 3 (ZOL+OZ500) with osteonecrosis induced according to the same protocol and local therapy with OZ (500 mEq/kg) at a concentration of 0.3 mg/kg for 2 min at baseline, two and four days post-surgery; group 4 (ZOL+OZ600), with osteonecrosis and local therapy using OZ (600 mEq/kg); and group 5 (ZOL+OZ700), with osteonecrosis and local application of OZ (700 mEq/kg) following the same protocols. The concentrations of ozonized sunflower oil chosen for the experimental groups were based on the clinical results of tissue repair in the systematic review by Lim et al. (2019) [26].

### 2.2. Outline of the Surgical Procedure

All experimental procedures that could involve pain or discomfort for the animals were performed under anesthesia with a combination of 50 mg/kg of intramuscular ketamine and 5 mg/kg of xylazine hydrochloride. They also received mepivacaine hydrochloride for local anesthesia and hemostasis of the operated area.

**Figure 1:**
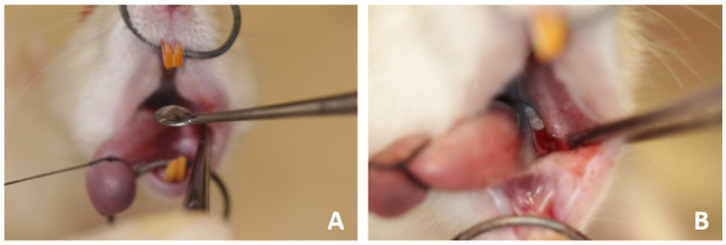
(**A**,**B**) Application of ozonized sunflower oil to the socket after tooth extraction.

The intervention started with the induction of experimental periodontitis, by placing a cotton bandage around the lower left first molar of each animal. This aimed to facilitate the extraction of these teeth, since a decrease in the supporting bone helps to avoid apical root fracture and the need for more invasive tooth extraction techniques (Figure 1).

Furthermore, periodontitis is a factor that leads to the induction of osteonecrosis [13]. After 3 weeks of applying ZOL medication, the rats were positioned on an operating table, where the cotton ligatures were removed, and syndesmotomy of the tissues adjacent to the tooth were performed, followed by the dislocation and extraction of the lower left first molars using dental surgical materials adapted to animals.

The animals of groups OZ500, OZ600 and OZ700 underwent ozone therapy by the application of 0.1 mL of ozonized oil using a 1 mL syringe applied into the post-extraction socket with a Molt 2/4 surgical curette. The oil was kept in place for 2 min before suturing the gingiva.

### 2.3. Euthanasia

The animals in groups SAL, ZOL, OZ500, OZ600, and OZ700 were euthanized 28 days post-extraction, totaling 7 weeks.

Euthanasia was performed by cardiac perfusion. The animals were sedated with the same anesthetic protocol used in surgical procedures, using a combination of 100 mL of saline solution and 0.1% heparin and 800 mL of 4% formaldehyde (Sigma Chemical^®^, St. Louis, MO, USA) in phosphate-buffered saline (PBS—Sigma Chemical^®^) 0.1 M, 4 °C, pH 7.4. Each animal received, on average, 500 mL of the solutions during cardiac perfusion.

### 2.4. Analyses

#### 2.4.1. Clinical Analysis

Immediately after sedation, prior to euthanasia, a clinical photo of each reparative socket was taken using a Canon D5i camera (Canon, Brazil) with circular flash and 105 mm macro lens, by an experienced operator (M.C.S.) always using the same calibration. The analyzed clinical characteristics were the color of the soft tissue covering the socket, its closure or not, as well as the presence of an open socket with or without exposed bone tissue, bone sequestration, and suppuration.

#### 2.4.2. Analysis and Processing of Calcified Tissues: Micro-Computed Tomography (Micro-CT)

Prior to the three-dimensional analysis of the bone tissue structure, the jaws of the animals from all groups were removed from the fixative and washed for 12 h in running water in order to remove any residue. Next, they were stored in 70% alcohol for transportation to the analysis site. The jaws were then X-rayed, and the images were uploaded to a digital microtomography system. The 6 µm thick sections were scanned using a SkyScan microtomography scanner (SkyScan 1176 Bruker Micro-CT, Aatselaar, Belgium, 2003) at 90 Kv and 111 µA, with a copper filter and rotation step of 0.05 mm. The areas responsible for the projection of X-rays onto the samples were stored and reconstructed, determining the area of interest using the program NRecon (SkyScan, 2011; Version 1.6. 6.0).

**Figure 2:**
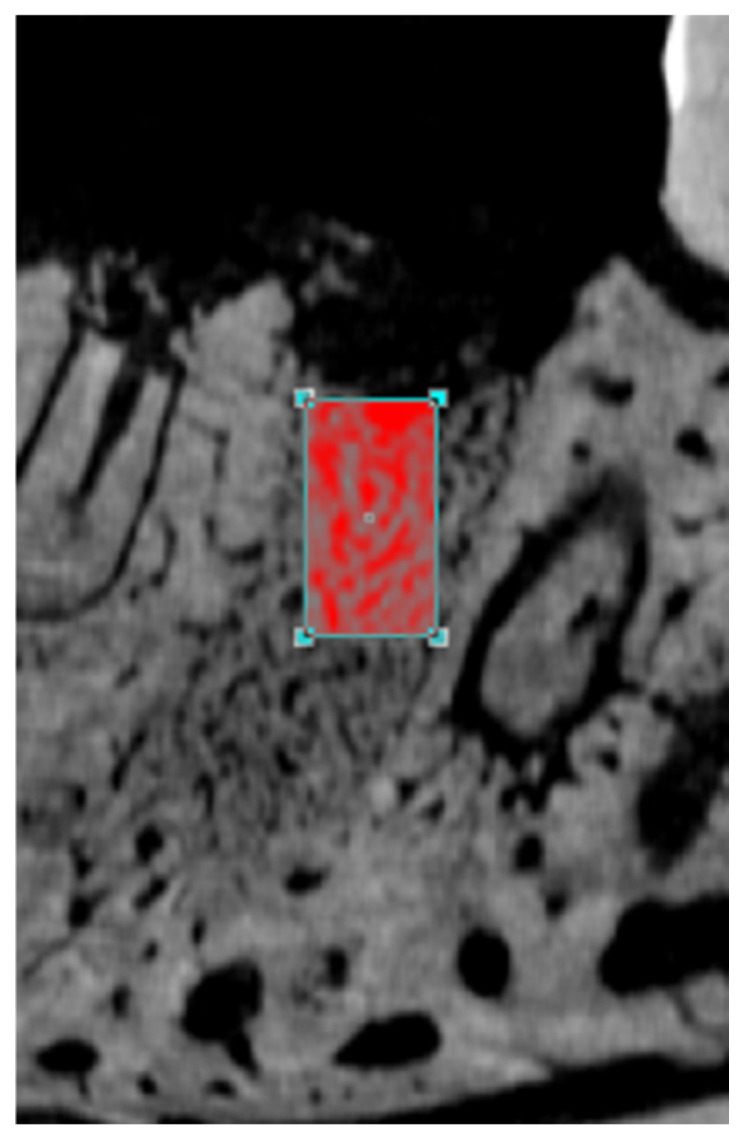
The radiographic area of interest (ARI) was defined as a 4 mm^3^ region at the site of the lower left first molar extraction.

The images were reconstructed in the program Data Viewer (SkyScan, Version 1.4.4 64-bit) to adapt the standard positioning for all samples, enabling observation in three planes (transversal, longitudinal, and sagittal). Then, using CTAnalyser—CTAn (2003-11SkyScan, 2012 Bruker Micro-CT Version 1.12.4.0), the radiographic area of interest (ARI) was defined, delimited by an area of 4 mm^3^ that includes the portion of the tooth extraction site previously occupied by the mesial and distal roots of the lower left first molar and adjacent tissues. The CTAn software analyzed and measured the image according to gray scales. After adjusting and removing the grey shades from the corresponding area of the socket, the threshold used in the analysis was 49–95 gray shades, which made it possible to obtain the bone volume inside the sockets (Figure 2).

The analyzed parameters refer to the quantity of bone tissue (BV = bone volume) and quality of bone tissue (Tb.Th = thickness of bone trabeculae, Tb.SP = separation of bone trabeculae, Tb.N = number of trabeculae and Po, tot = percentage of total porosity).

#### 2.4.3. Laboratory Processing of Parts

Following microtomography, the hemi-mandibles were included in paraffin and submitted to microtomy in 5 µm cuts, in accordance with previous studies [9,27]. Histological sections were obtained from the lingual to the buccal portion of the sockets and stained with hematoxylin and eosin (HE).

#### 2.4.4. Microscopic Analysis and Microscopic Area of Interest (AMI)

**Figure 3:**
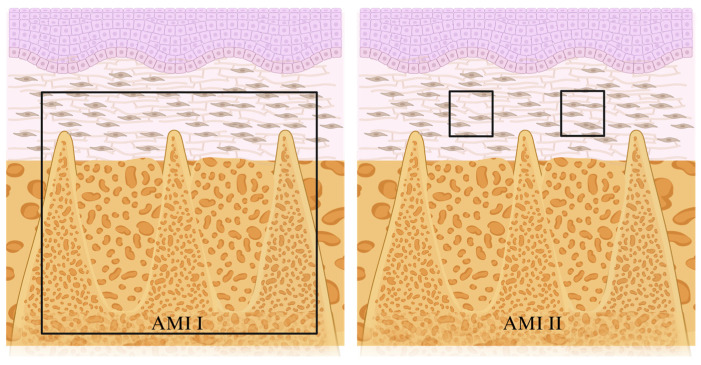
AMI (I) and AMI (II). The AMI (I) consisted of a 4 mm × 4 mm area that included the portion of the tooth extraction site previously occupied by the mesial and distal roots of the lower left first molar and adjacent tissues. The AMI (II) consisted of two areas of 250 × 250 μm located in the connective tissue covering the tooth extraction site.

The microscopic analyses were performed by a certified histologist blinded to experimental groups (E.E.). Three histological sections from the buccal, middle, and lingual portion of the tooth extraction sites were submitted to histometric analysis. The area of interest was divided in two [9]: AMI (I) and AMI (II), according to the performed microscopic analysis (Figure 3). The AMI (I) consisted of a 4 mm × 4 mm area that included the portion of the tooth extraction site previously occupied by the mesial and distal roots of the lower left first molar and adjacent tissues. Its distal limit was composed of a line located parallel to the coronal dentin and the root surface of the lower left second molar, extending 4 mm mesially. Its coronal limit was composed of a line parallel to the limit of the cementogingival margin of the lower left second molar, extending 4 mm apically. The AMI (II) consisted of two areas of 250 × 250 μm located in the connective tissue covering the tooth extraction site. The limit of the areas was determined by a line located in the center of the connective tissue, perpendicular to the long axis of the teeth which divides this tissue in the coronoapical direction. Two other lines were used, one parallel to the central portion of the tooth extraction site previously occupied by the mesial root, and another parallel to the central portion of the tooth extraction site previously occupied by the distal root of the first molar. The intersection of these lines determined the center of the two analyzed areas.

#### 2.4.5. Bone Tissue Histometry: Neoformed Bone Tissue (NFBT) and Non-Vital Bone Tissue (NVBT)

The histometric analysis of the neoformed bone tissue (NFBT) was performed using photomicrographs of the areas adjacent to the socket, captured with the aid of image analysis software (Axiovision 4.8.2^®^ Carl Zeiss, Göttingen, Germany). The area occupied by NFBT was established and expressed in μm^2^ as mean ± standard deviation in each experimental group. In the AMI (I), images were captured using a digital camera (AxioCam^®^ Carl Zeiss, Gottingen, Germany) coupled to a light microscope (AxioLab^®^) and connected to a microcomputer. The total amount of bone tissue was calculated using image analysis software (Axiovision 4.8.2^®^ Carl Zeiss). Subsequently, the percentages of NFBT and non-vital bone tissue (NVBT) were calculated using the same program. The NVBT represents a region where more than ten neighboring osteocyte lacunae are either empty or contain remains of necrotic osteocytes [9]. The captured regions are as follows: (1) extraction site previously occupied by the mesial root of the lower first molar; (2) extraction site previously occupied by the distal root of the lower first molar; (3) bone tissue located in the inter radicular septum and (4) bone tissue located in the edentulous area located immediately in front of the lower first molar.

Regions 1 and 2 were used to evaluate the percentage of NFBT in the alveoli. To this end, two rectangles were positioned, one in the center of the mesial root extraction site and another in the center of the distal root extraction site of the lower first molar. The largest base of such rectangles followed the long axis of the roots. The rectangles had the base of the sockets as the apical limit.

Regions 3 and 4 were used to measure the percentage of NVBT in the vicinity of the tooth extraction site. Two rectangles were positioned, one in the center of the interradicular septum, with the largest base of this rectangle following the long axis of the roots, and another in the bone tissue located in the edentulous area immediately in front of the extraction site, which was previously occupied by the mesial root of the lower first molar, with the largest base of the rectangle accompanying the alveolar bone crest. Each of these rectangles was 710 μm high and 530 μm wide, making up an area of 0.38 mm². Since the extraction site and surrounding areas were evaluated in four regions, the total area amounted to 1.52 mm [2,3,4,5,6,7,8,9,10,11,12,13,14,15,16,17,18,19,20,21,22,23,24,25,26,28,29].

#### 2.4.6. Anatomopathological Analysis of Metabolizing Organs

To verify the potential toxicity of the peroxides released by the ozonized oil, metabolizing organs (lungs, liver, and kidney) were collected and included in paraffin for laboratory processing. The pieces were microtomized into 5 µm thick sections and HE stained. Histopathological analysis was then performed with an optical microscope (Zeiss, Axioshop, Germany), in order to verify cellular infiltration, hyperplastic tissues, metaplastic or dysplastic transformations [30].

### 2.5. Statistical Analysis

All quantitative data were analyzed using SigmaPlot 12.0 (Exakt Graphs and Data analysis, San Jose, CA, USA) at a significance level of 5%. The microtomographic and histometric parameters were subjected to the normality test (Shapiro–Wilk) and the Kruskal–Wallis analysis of variance test, followed by the Student–Newman–Keuls post hoc test when *p* < 0.05.

## 3. Results

### 3.1. Clinical Analysis

**Figure 4:**
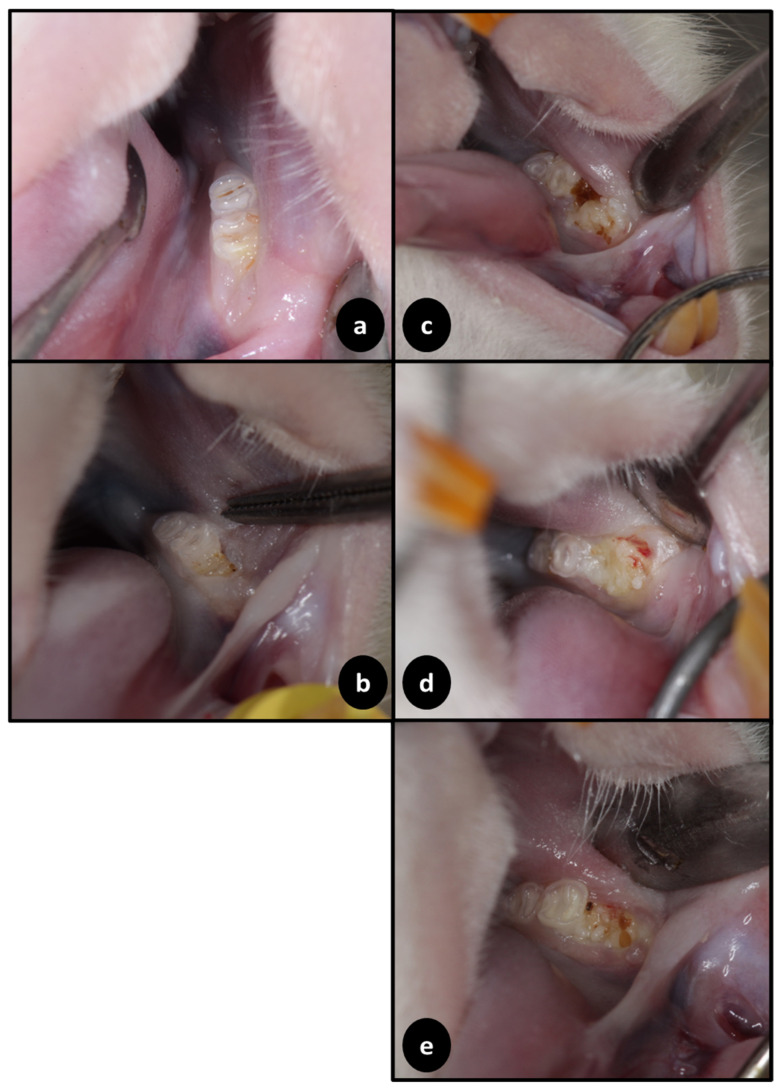
Clinical appearance of the groups SAL, ZOL, ZOL+OZ500, ZOL+OZ600, and ZOL+OZ700, 28 days after extraction of the lower left first molar. (**a**) SAL group: alveolar process with attached gingiva and no signs of infection. (**b**) ZOL group: alveolar process with bone exposure, without soft tissue covering. (**c**) ZOL+OZ500 group: alveolar process with no signs of infection or exposed bone. (**d**) ZOL+OZ600 group: alveolar process with delayed repair, without signs of infection or bone exposure, and a minor bleeding point. (**e**) ZOL+0Z700 group: alveolar process with slight edema in the region of attached gum and delayed tissue repair.

It should be noted that in some of the photographs presented below (Figure 4), yellow-brownish food particles can be seen in the regions of the reparational sockets.

In the ZOL+OZ500 group, it was possible to observe specimens with a delayed alveolar repair process in the lower left first molar region, albeit without bone exposure or signs of infection. In the ZOL+OZ600 group, in addition to a delayed alveolar repair process, a certain edema was seen in the attached gum region, but also without exposed bone tissue or signs of infection. Finally, in the ZOL+OZ700 group, animals with a delayed alveolar repair process were also observed, equally without bone exposure or signs of infection.

### 3.2. Micro-CT

In terms of bone volume (BV), the ZOL+OZ600 group showed a higher value than the other groups, followed by the ZOL+OZ700 and ZOL+OZ500 groups (*p* < 0.05), with these groups outperforming the control group (Table 1). For Tb.N (trabecular number), the SAL-treated group had a higher value compared to the ZOL+OZ500 and ZOL+OZ700 groups (*p* < 0.05), followed by the ZOL+OZ600 group, indicating a more trabeculated bone structure (Table 2). Regarding Tb.Sp, which analyzes trabecular separation, the ZOL+OZ500 group had the highest separation, followed by the ZOL+OZ600 group, both showing a statistically significant difference compared to the ZOL group (*p* < 0.05) (Table 2). For the parameters Tb.Th (trabecular thickness) (Table 2) and Po(tot) (porosity) (Table 1), all groups presented similar results (*p* > 0.05).

### 3.3. Histological Analysis

**Figure 5:**
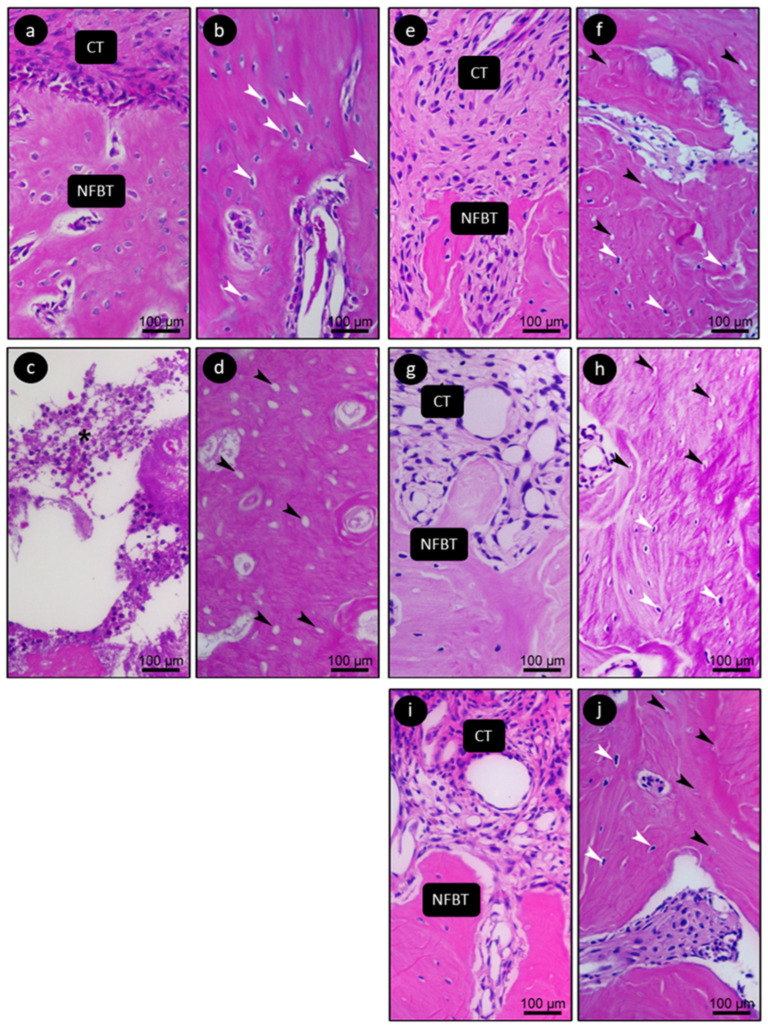
Histological appearance of the dental extraction site and surrounding areas, 28 days postoperatively. Photomicrograph showing the histological appearance of the bone tissue and connective tissue at the tooth extraction site 28 days postoperatively in groups (**a**) SAL, (**c**) ZOL, (**e**) ZOL+OZ500, (**g**) ZOL+OZ600, and (**i**) ZOL+OZ700. Photomicrograph showing the histological appearance of the bone tissue located in the immediate vicinity of the tooth extraction site 28 days postoperatively in groups (**b**) SAL, (**d**) ZOL, (**f**) ZOL+OZ500, (**h**) ZOL+OZ600, and (**j**) ZOL+OZ700. White arrows: osteocytes; ct: connective tissue; NFBT: neoformed bone tissue. Original magnification: 400×. Scale bars: 100 µm. Hematoxylin and eosin (HE) staining. The white arrows show the presence of osteocytes and the black arrows show the gaps of empty osteocytes.

Figure 5 shows a representation of the histological characteristics of the dental extraction sites and their surroundings at 28 days postoperatively in the animals of their respective groups.

In the SAL group, the tooth extraction area showed a large amount of NFBT, as well as a presence of osteocyte lacunae in the extracellular matrix (white arrows) in the bone adjacent to the extraction site. This was not the case in the ZOL group, which showed few areas of NFBT, and the region of extracellular matrix showed NFBT with osteocytes both in NFBT and the adjacent bone. Most slides reveal an intense inflammatory process, suggestive of bacterial hyphae. The experimental groups (OZ500, OZ600 and OZ700) showed NFBT with osteocytes both in NFBT and the adjacent bone, as well as overlying mature connective tissue (CT).

### 3.4. Pathology of Metabolizing Organs

**Figure 6:**
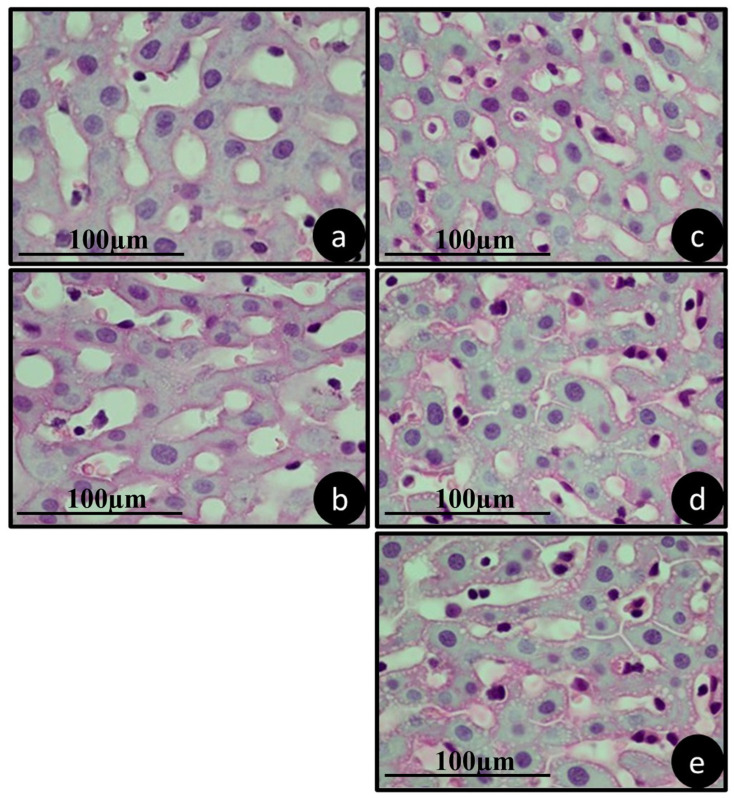
Representative histology of the livers of animals from groups (**a**) SAL, (**b**) ZOL, (**c**) ZOL+OZ500, (**d**) ZOL+OZ600, (**e**) ZOL+OZ700, collected during euthanasia 28 days after extraction of the first molars. HE stained, 1000× magnification, scale 100 µm.

**Figure 7:**
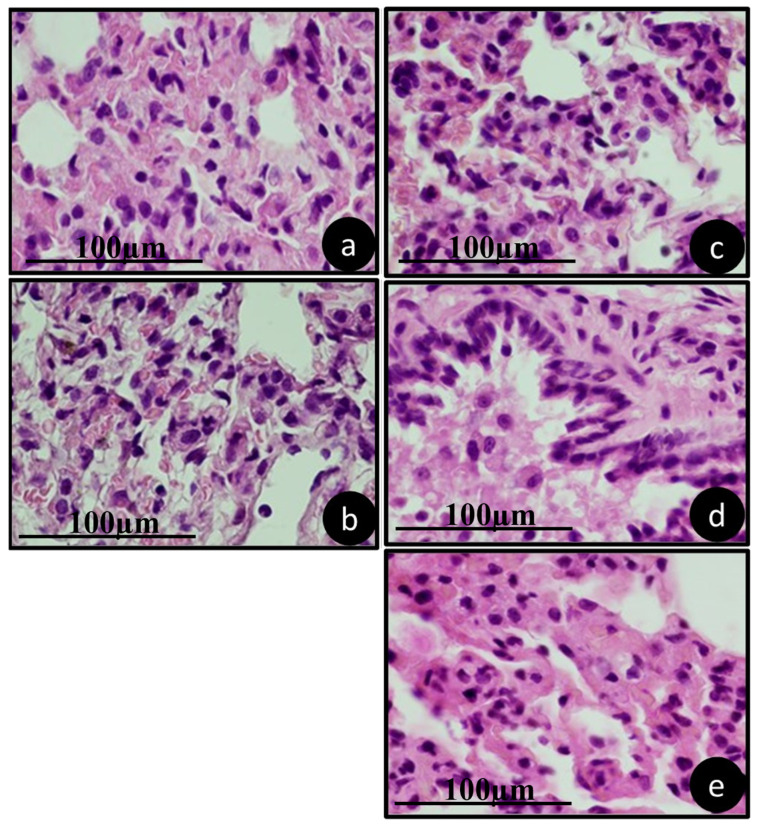
Representative lung histology of animals in groups (**a**) SAL, (**b**) ZOL, (**c**) ZOL+OZ500, (**d**) ZOL+OZ600, (**e**) ZOL+OZ700, collected during euthanasia 28 days after first molar extraction. HE stained, 1000× magnification, scale 100 µm.

**Figure 8:**
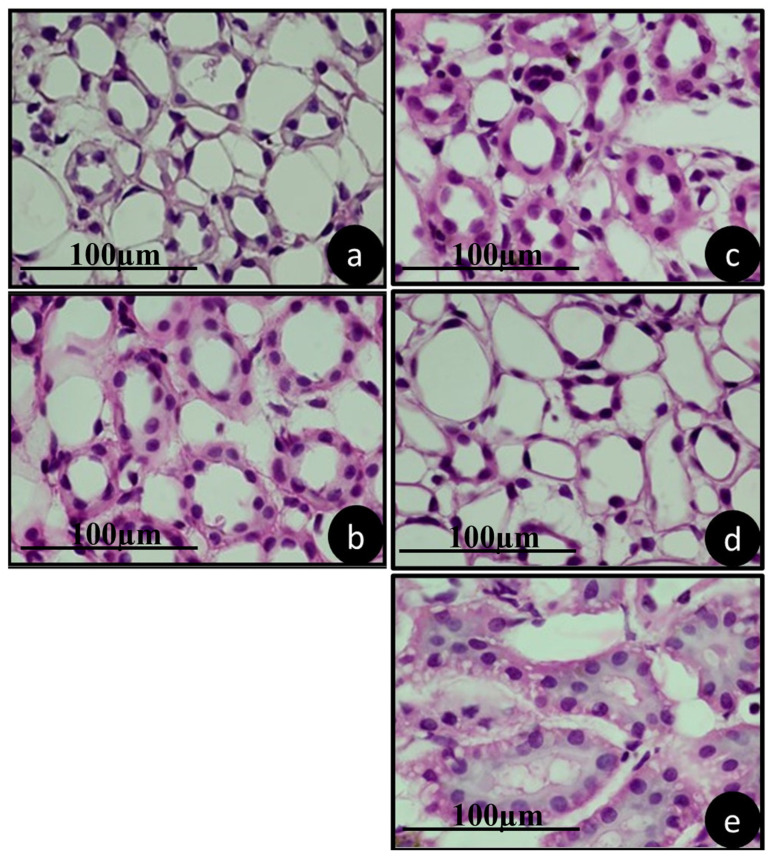
Representative kidney histology of animals in groups (**a**) SAL, (**b**) ZOL, (**c**) ZOL+OZ500, (**d**) ZOL+OZ600, (**e**) ZOL+OZ700, collected during euthanasia 28 days after first molar extraction. HE stained, 1000× magnification, scale 100 µm.

Figure 6, Figure 7 and Figure 8 show a representation of the histological characteristics of the metabolizing organs. It is evident that the therapies used did not cause changes in the morphology and histology of the analyzed organs.

The livers presented hepatocytes and hepatic lobules of an organization and structure within the normal range and all fundamental elements present (Figure 6).

The lungs were structurally within normal limits, with alveolar structure, presence of cells and blood vessels (Figure 7).

The kidneys showed normal renal corpuscles and renal cells of an organization and distribution within the normal limits (Figure 8).

### 3.5. Bone Tissue Histometry: NFBT and NVBT

**Figure 9:**
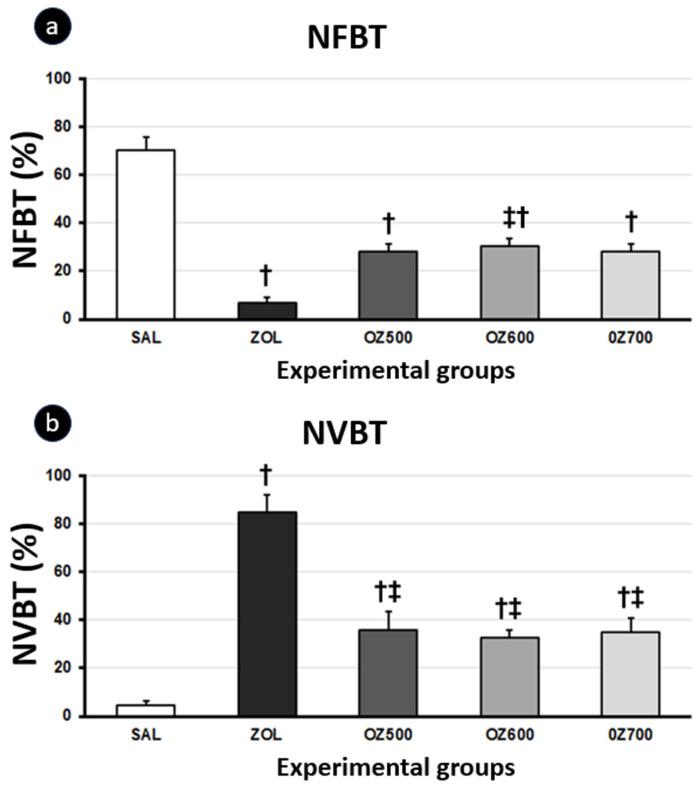
(**a**) Percentage of NFBT and (**b**) non-vital bone tissue (NVBT) at the tooth extraction site 28 days post-extraction. Statistical tests: Kruskal–Wallis analysis of variance and Student–Newman–Keuls post hoc test. †: statistically significant difference in relation to the SAL group; ‡: statistically significant difference in relation to the ZOL group.

The histometric analysis of NFBT showed values with statistically significant differences in all groups compared to the SAL group, which had more NFBT (*p* < 0.05). When comparing therapies that used ZOL, the ZOL+OZ600 group had more NFBT, followed by OZ500, OZ700, and ZOL. With reference to NVBT, all groups showed a difference in comparison with the SAL group (*p* < 0.05). However, the three experimental groups also showed a difference in relation to the ZOL group (*p* < 0.05) (Figure 9).

## 4. Discussion

The occurrence of MRONJ has increased in recent years, due to the high rate of prescription of antiresorptive drugs [2,21], which can be administered intravenously or orally. According to the American Association of Oral and Maxillofacial Surgeons (AAOMS) (2022), in cases of bisphosphonate usage in the treatment of osteoporosis, the risk of developing MRONJ is 0.02% to 0.05%. For oncological applications, the risk increases to 1.3–1.8% after three years of using this therapy [2,21,31]. At present, workshops, clinical research and task forces are seeking to generate new knowledge on the subject. These efforts have already led to a significant evolution in the understanding of medication-related osteonecrosis of the jaw.

Regardless of any indications for use, the duration of antiresorptive therapy certainly appears to be a relevant factor in the development of MRONJ, characterizing yet another element in its multifactorial etiopathogenesis [32,33]. Therefore, especially in the case of bisphosphonates, which have a half-life of years due to their high affinity for hydroxyapatite, interrupting the short-term administration of the medication is not beneficial to the patient. Additionally, it may not alleviate the symptoms due to this medication’s pharmacokinetics [20,31].

Hence, the search for adjuvant preventive therapies to manage MRONJ becomes important, in particular, studies that simulate a predominant clinical condition in this scenario, such as senescence. Moreover, knowledge about the systemic conditions that can increase MRONJ incidence is an important factor in its management, taking into account the significance of preventive therapeutic strategies [20,31]. Khan et al. (2015) emphasized the importance of improving quality of life, and that the search for preventive therapies has a positive impact on the clinical prognosis [34].

Several publications have already elaborated on the high antioxidant capacity of ozone. According to Lim et al. (2019) ozone releases free oxygen radicals, which increase the phagocytic capacity of immune cells and assist in wound healing [21,26]. The release of free radicals becomes extremely important in a microenvironment under hypoxic conditions, as is the case with MRONJ, where tissue with an oxygenation deficiency is unable to meet the demands necessary for the repair process to occur. Furthermore, it is known that such characteristics favor an increase in local vascularization by inducing the production of growth factors, something that is also in deficit in patients who use bisphosphonates [20]. Against this background, the present research has sought to analyze whether local therapies with ozonized oil have the potential to mitigate the deleterious effects of zoledronate, particularly in cases of hypoxia of the post-exodontic reparative tissues in senescent rats and with the administration of an oncological dose of zoledronate.

For all OZ concentrations used in this study, tissue maintenance in the socket was noted. Extensive areas with osteocytes were identified in the extracellular matrix, representing vital bone tissue. These findings corroborate the study by Monteiro et al. (2021), which identified similar histological regions and showed promising preclinical results for the use of OZ in MRONJ prevention [20]. It is important to consider that the choice for senescent rats aged 18 months with a post-exodontic site in the posterior region of the mandible stems from our search for the best conditions to approximate clinical practice. Furthermore, the SAL group is like a sham, related to using the same solution for the ZOL titulation. Thus, all groups underwent the same level of stress. In such a critical situation, it is believed that reparative responses in animals can be analyzed more accurately and, posteriorly, put into clinical practice. Additionally, this research has compared three concentrations of OZ dispersion in order to assess which one is more appropriate for clinical treatments.

Ripamonti et al. (2011) presented a clinical applicability of ozone in oily solution in cases of MRONJ with small bone exposures, not associated with surgical intervention. Their preliminarily observation was that the therapeutic use of this solution is a safe and simple alternative to manage the lesions, in addition to being low cost and self-administrable by patients [21]. Results from a preliminary clinical study focused on analyzing oral conditions and assessing factors such as bone sequestration, infection, and healing time. However, in vivo studies remain essential, as they enable a deeper evaluation and understanding of the repair processes and biological behavior. It is important to keep in mind that it is essential to understand how these treatments are effective in a controlled environment, such as in an animal investigation.

The antimicrobial and modulating action of OZ repair involves the release of reactive oxygen species, resulting from oxidative stress. Although this mechanism has been described in the literature, there is still no protocol for the use of this therapy with regard to the concentration of peroxides and number of applications to maximize effectiveness [20,22]. Moreover, the absence of any standardization of the solutions makes it difficult to compare these studies. The present study employed an OZ certified by the company with biochemical analysis, proving that the concentration used corresponds to what was applied in the area. Thus, it was possible to identify the physicochemical properties of the oil production.

Previously published works noted that high doses of OZ, in the order of ~3000 mEq/kg, delayed the healing of skin wounds in mice [35,36]. The concentrations used in this study are at most 700 mEq/kg, which, in addition to not approaching this possible tissue overdose, did not have any deleterious effects on the tissue of the post-exodontic reparative socket or on the metabolizing organs—and therefore seemingly quite safe for clinical use. Given these findings, it was possible to verify that the three studied concentrations achieved vital bone formation with the entrapment of osteocytes in the extracellular matrix. In MRONJ conditions, filling the post-exodontic socket with vital neoformed bone is essential. As such, this preliminary study has led us to believe that the concentration of OZ+600 is ideal for both the prevention and treatment of MRONJ. In future studies, the prospective monitoring of patients undergoing these therapies may guide clinical management aimed at reestablishing the morphology and health of oral tissues.

In addition to biological information on the repair of bone tissue in the post-exodontic socket in senescent rats that were administered an oncological dose of a potent anti-resorptive, closure of the socket by soft tissues is essential. Therefore, the clinical photographs presented in this study are crucial for understanding how the applied therapies enabled the recovery of the post-exodontic socket without the infections or exposed bone tissue that could be a continuation of MRONJ characteristics, as is evident in the ZOL group. The OZ+600 group showed areas with slight edema and areas with bleeding points. It is important to note that the trauma caused by food fragments during animal feeding, which can be seen in some of the pictures, showed neo-angiogenesis in the covering soft tissues, a property of OZ that had previously been reported [15,27].

In several countries, the use of ozone therapy is still regulated, while in others, it is restricted regardless of the administration route. This results from a lack of clinical evidence for OZ use, which is why further controlled clinical studies and investigations that prove the biological behavior justifying its clinical use are needed. It is imperative that, prior to any controlled clinical investigations, the biological behavior is investigated. To this end, pre-clinical studies using a physiological organism (e.g., animals) are fundamental. Notwithstanding the limitations of the animal model, like differences in reparative behavior and metabolism times, the analysis conducted here has shown that the dispersion of ozonized oil in the given concentrations provides a solid basis for future clinical studies.

Another aspect to be discussed is the safety of using local therapies like OZ compared to application in the form of local gas or systemic application, as well as in association with systemic antimicrobial medications that can increase tissue antioxidants, such as antibiotics and pentoxifylline. Although the animal study presents some of the local conditions of MRONJ and, in clinical speculation, the administration of these medications is necessary, safe therapy with OZ has proven itself as an adjuvant in the re-establishment of reparative tissues.

With reference to the need for investigations in animal models in contrast to clinical research on MRONJ, these provide us with results about the symptoms and characteristics of tissue closure. However, it is difficult to observe biological responses regarding tissue repair, which is fundamental for the establishment of guidelines for clinical application.

The obtained results will serve as a scientific basis for future controlled clinical studies using OZ in the treatment of MRONJ and other conditions related to tissue damage. As this is a pioneering study, there is great potential for future research on other experimental populations, different systemic situations, and associations with other methods of using ozone therapy, in addition to comparisons with other local therapies to treat MRONJ.

## 5. Conclusions

The results of this study suggest that ozone therapy in an oily solution, at concentrations of 500, 600, and 700 mEq/kg, may help prevent MRONJ by enhancing tissue quality and particularly promoting osteogenesis in healing sockets. The most notable effects were observed in the ZOL+OZ600 group following extraction in senescent rats treated with a high dosage of zoledronate (ZOL).

## Figures and Tables

**Table 1:** Microtomographic data (mean ± standard deviation) for quantitative analysis of the bone tissue (BV and Po.Tot).

Groups	BV (mm)	Po.Tot (%)
**SAL**	0.35 ± 0.07	61.12 ± 4.62
**ZOL**	0.03 ± 0.01	54 ± 3.43
**ZOL+OZ500**	0.25 ± 0.13 b	52.5 ± 3.13
**ZOL+OZ600**	0.39 ± 0.05 abc	61.21 ± 4.45
**ZOL+OZ700**	0.24 ± 0.05 abd	57.85 ± 9.9

Means followed by different lowercase letters show statistical difference between the groups within each dependent variable (*p* < 0.05, ANOVA followed by Tukey HSD test). The letters a, b and c represent statistical differences between groups.

**Table 2:** Microtomographic data (mean ± standard deviation) in mm for qualitative analysis of the bone tissue (Tb.N, Tb.Sp, and Tb.Th).

Groups	Tb.N (1/mm)	Tb.Sp (mm)	Tb.Th (mm)
**SAL**	5.301 ± 4.47	1.9 ± 4.62	0.09 ± 0.01
**ZOL**	3.46 ± 9.02	54 ± 3.43 a	0.09 ± 0.08
**ZOL+OZ500**	2.29 ± 7.36 a	52.50 ± 3.13 b	0.102 ± 0.01
**ZOL+OZ600**	3.95 ± 8.86	61.21 ± 4.45 b	0.10 ± 0.01
**ZOL+OZ700**	2.19 ± 7.84 a	57.85 ± 9.90	0.11 ± 0.02

Means followed by different lowercase letters show statistical difference between the groups within each dependent variable (*p* < 0.05, ANOVA followed by Tukey HSD test). The letters a, b and c represent statistical differences between groups.

## Data Availability

All data supporting the findings of this study are available within the paper and its the methodology used in this study is detailed and described on DOI: 10.1089/ten.TEC.2023.0159.
